# Characterization of the complete mitochondrial genome of the predatory mite *Stratiolaelaps scimitus* (Acari: Laelapidae)

**DOI:** 10.1080/23802359.2020.1717393

**Published:** 2020-01-27

**Authors:** Yi Yan, Na Zhang, Xinran Wu, Kai Liu, Chenglin Liu, Lixia Xie

**Affiliations:** Department of Entomology, College of Plant Protection, Shandong Agricultural University, Shandong Provincial Key Laboratory for Biology of Vegetable Diseases and Insect Pests, Taian, China

**Keywords:** Mesostigmata, gene order, mitochondrial genome, phylogeny, Dermanyssoidea

## Abstract

In this study, we recovered the complete mitochondrial genome of *Stratiolaelaps scimitus* through Illumina sequencing data. The circularized mitogenome is 16,009 in length, which consists of 37 genes (13 protein-coding genes, 22 transfer RNA genes, and 2 ribosomal RNA genes). The overall base composition is 35.4% for A, 40.1% for T, 16.8% for G, 7.7% for C, demonstrating an extreme bias of high AT content (75.5%). The whole mitogenome of *S. scimitus* and other Acari mitogenomes (10 species, in total) were used for phylogenetic analysis, and the result showed that the relationship of *S. scimitus* was close to *Varroa destructor* in the same Superfamily Dermanyssoidea.

Predatory mites play the leading role in commercial augmentative biological control. *Stratiolaelaps scimitus* Womersley (Acari: Mesostigmata: Laelapidae) is a polyphagous soil-inhabiting predatory mite, which is widely marketed for its use in greenhouse production systems to manage populations of dark-winged fungus gnats and control of soil-pupating thrips (Cabrera et al. 2005; Sun et al. 2018). Besides, *S. scimitus* has been studied for its potential as a biocontrol agent against other edaphic pests (Xie et al. 2018). To date, however, only eight complete mitogenomes of Mesostigmata have been reported (NCBI, accessed 2019 Dec 18). For further development of biocontrol strategies using the advance evolutionary studies, we report the complete mitochondrial genome of *S. scimitus* Womersley, representing the first mitogenome in Laelapidae.

In this study, the voucher specimens of *S*. *scimitus* were collected from topsoil under the bamboo of Shandong Agricultural University, Taian, Shandong, China (36.114°N, 117.064°E), and reared for more than 15 generations, the acarid mite *Tyrophagus putrescentiae* (Acari: Acaridae) as prey. Rearing of these predatory mites and the prey were conducted at 25 ± 1 °C, 80 ± 5% R.H. in dark in Shandong Agricultural University. Pooled male and female mites were stored in absolute ethanol (NCBI BioSample accession SAMN13425621; voucher specimen number 190609SC). Voucher specimens and DNA were deposited in Tianjin Novogene Bioinformatics Technology Co., Ltd, China. The mitogenome was sequenced on an Illumina platform and assembled using NOVOPlasty v2.7.2 (Dierckxsens et al. 2017), annotated with MitoZ v2.3 (Meng et al. 2019), and deposited in the GenBank with the accession number MN781133.

The circularized mitogenome is 16,009 bp in length, which consists of 37 genes (13 protein-coding genes, 22 transfer RNA genes, and 2 ribosomal RNA genes). The overall base composition is 35.4% for A, 40.1% for T, 16.8% for G, 7.7% for C, demonstrating an extreme bias of high AT content (75.5%). Nine PCGs, 12 tRNA genes, and one rRNA genes were located on the positive strand, while four PCGs (ND1, ND4, ND4L, and ND5), 10 tRNA genes (tRNA^Phe^, tRNA^His^, tRNA^Leu^, tRNA^Leu^, tRNA^Gln^, tRNA^Tyr^, tRNA^Pro^, tRNA^Ser^, tRNA^Val^, and tRNA^Cys^), and one rRNA genes (l-rRNA) were located on the reverse strand. In addition, the mitochondrial gene order is known to be highly variable in Acari compared with the ancestral pattern of gene arrangement to arthropods (Xue et al. 2016). Compared with *Varroa destructor* (NC_004454.2), the gene located between COX1 and COX2 changed to tRNA^His^. The position of tRNA^Phe^, ND5, ND4, and ND4L exchanged with the combination of tRNA^Thr^, ND6, and CYTB. Besides, tRNA^Ser^ exchanged with tRNA^Val^.

Amino acid sequences were aligned using MAFFT v7.407 (Katoh and Standley 2013) and trimmed with trimAl v1.4.1 (Capella-Gutiérrez et al. 2009) with the heuristic method ‘automated1’. The phylogeny was reconstructed by IQ-TREE v1.6.10 (Nguyen et al. 2015) under the LG + C20 + F + G model. And, the tree topology was verified under 1000 bootstrap. The whole mitogenome of *S. scimitus* and other Acari mitogenomes (10 species, in total) were used for phylogenetic analysis, and the result showed that the relationship of *S. scimitus* was close to *V. destructor* in the same Superfamily Dermanyssoidea (Figure 1).

**Figure 1.**
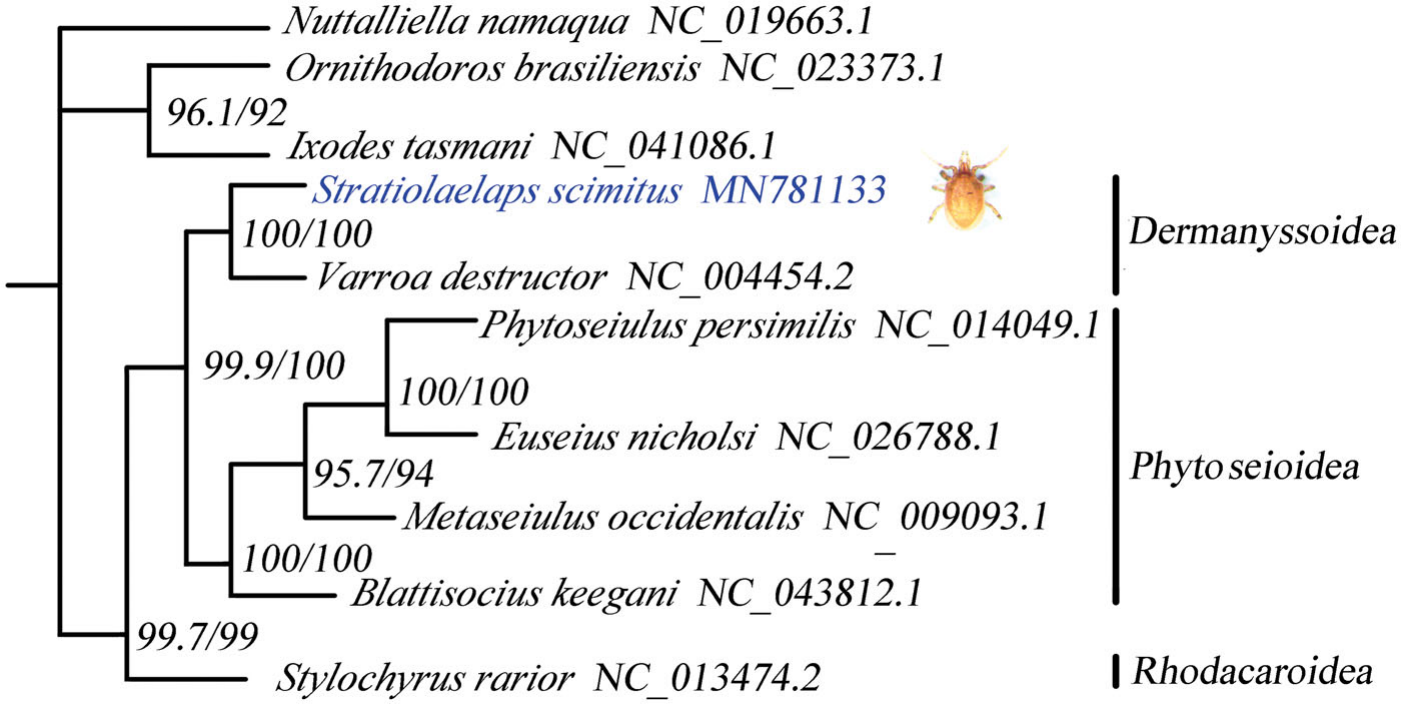
Phylogenetic tree inferred from 13 PCGs. SH-aLRT and UFBoot support values are given on nodes.
